# Lymphocyte Levels and Morbidity and Mortality in Cardiovascular Surgery With Cardiopulmonary Bypass

**DOI:** 10.21470/1678-9741-2023-0136

**Published:** 2024-07-15

**Authors:** Renata Costa Café de Castro, Paula Natividade Costa, Eduardo Augusto Victor Rocha, Isabella Victoria da Cunha Peixoto Ribeiro, Maria Paula Parreira

**Affiliations:** 1 Faculdade Ciências Médicas de Minas Gerais, Belo Horizonte, Minas Gerais, Brazil; 2 Department of Cardiac Surgery, Hospital Universitário Ciências Médicas, Belo Horizonte, Minas Gerais, Brazil

**Keywords:** Cardiovascular Surgical Procedures, Lymphocyte Count, Indicators of Morbidity and Mortality

## Abstract

**Introduction:**

A year ago, in a sample of 113 patients, our research group found that a high number of lymphocytes in the immediate postoperative period was correlated to a poor prognosis in cardiovascular surgeries. This study is an expansion of the initial study in order to confirm this finding.

**Methods:**

We analyzed the data of 338 consecutive patients submitted to cardiovascular surgeries with cardiopulmonary bypass performed at Hospital Universitário Ciências Médicas (Belo Horizonte/Brazil) from 2015 to 2017. We analyzed 39 variables with the outcomes death, hospital stay, and intensive care unit stay.

**Results:**

The value of lymphocytes in the immediate postoperative period > 2175.0/mm³ was an indicator of poor prognosis in this sample (*P*<0.001). The variables female sex, age, high level of European System for Cardiac Operative Risk Evaluation II, increased stay in the intensive care unit and in the ward, elevation of creatinine in the preoperative period and at intensive care unit discharge, elevation of the percentage of immediate postoperative period segmented neutrophils, high immediate postoperative period neutrophil/lymphocyte ratio, fasting hyperglycemia, preoperative critical condition, reintubation, mild or transient acute renal failure, surgical infection, cardiopulmonary bypass, and aortic cross-clamping and mechanical ventilation durations also had an impact on the mortality outcome.

**Conclusion:**

The value of lymphocytes in the immediate postoperative period > 2175.0/mm^3^ was an indicator of poor prognosis in cardiovascular surgery with cardiopulmonary bypass.

## INTRODUCTION

Cardiovascular surgeries (CVS) have a great positive impact on survival and on restoring the functional capacity of patients, improving quality of life^[1]^. In Brazil, myocardial revascularization and valve replacement are the most frequent surgeries among adults^[1]^.

In view of the high frequency of these procedures, their costs, and high morbidity and mortality, some models of risk stratification in CVS were developed, such as the European System for Cardiac Operative Risk Evaluation (EuroSCORE) II and the Society of Thoracic Surgeons Score^[2,3,4]^. These models establish scores based on factors identified as predictors of death or complications^[4]^.

Mortality in cardiac surgeries has great variability, depending on the hospital, the volume of surgeries of the service studied, and the type of procedure performed^[5]^. In Brazil, due to its particularities, there is a need to improve the understanding of risk factors, and one of the ways to achieve this is to study the variables of procedures in the country^[6]^.

This research group conducted a retrospective observational cohort study a year ago with 113 patients undergoing CVS at the Hospital Universitário Ciências Médicas (Belo Horizonte/Brazil)^[7]^. We found that patients who evolved worse had an increased lymphocyte value in the immediate postoperative period (IPO)^[7]^. We followed this line of research in this study, seeking to expand the sample and validate the finding that is little studied. In addition, we analyzed other variables comparing them with outcomes such as death, hospital stay, and stay in an intensive care unit (ICU).

## METHODS

A retrospective observational study was conducted collecting data from the database of a consecutive series of patients operated at the Hospital Universitário Ciências Médicas from January 2015 to December 2017. We included all patients over 18 years of age who presented complete data in the medical records. Patients under the age of 18 years and who did not have complete data making it impossible to calculate EuroSCORE II were excluded. The final sample included 338 patients. Among these, some did not have all the researched variables in their medical records (because of intraoperative death or due to missing data), but this lack of data did not prevent the analysis of other registered data or the calculation of EuroSCORE II. The sample in each variable is demonstrated in Table 1. The patients did not receive any corticosteroid drugs preoperatively, intraoperatively, or postoperatively.

**Table 1:** Comparison of variables with the occurrence of death.

Variable	Death		*P*-value
	Yes	No	
Sex			0.003Q
Female	30 (57.7)	100 (35.0)	
Male	22 (42.3)	186 (65.0)	
Age, years	62.2 ± 12.6	57.9 ± 13.5	0.028M
	62.0 (53.8 – 72.0)	59.0 (50.0 – 68.0)	
EuroSCORE II	7.6 ± 13.5	2.6 ± 2.7	< 0.001M
	2.9 (2.0 – 5.5)	1.8 (1.0 – 2.9)	
EuroSCORE II risk mortality			< 0.001Q
Low	28 (53.8)	219 (76.6)	
Medium	12 (23.1)	44 (15.4)	
High	12 (23.1)	23 (8.0)	
Morbidity (> 7 days of hospitalization or > 4 days in ICU) (n=331)			0.003Q
Yes	23 (51.1)	212 (74.1)	
No	22 (48.9)	74 (25.9)	
Preoperative creatinine (n=333)	1.3 ± 0.9	1.0 ± 0.8	0.005M
	1.1 (0.9 – 1.3)	0.9 (0.8 – 1.1)	
Creatinine at ICU entry (n=322)	1.2 ± 0.9	0.9 ± 0.6	0.003M
	0.9 (0.7 – 1.4)	0.8 (0.7 – 1.0)	
Creatinine at ICU discharge (n=304)	2.0 ± 1.7	0.9 ± 0.5	< 0.001M
	1.4 (0.8 – 2.3)	0.8 (0.7 – 1.0)	
Postoperative lymphocytes (n=322)	2175.0 ± 1481.5	1447.1 ± 804.2	< 0.001M
	2012.5 (1179.2 – 2872.0)	1244.6 (893.6 – 1830.6)	
Postoperative lymphocytes (%) (n=322)	14.1 ± 6.7	10.8 ± 4.8	< 0.001M
	13.0 (10.0 – 20.0)	10.0 (7.0 – 14.0)	
Postoperative segmented neutrophils (%) (n=322)	76.1 ± 8.0	78.3 ± 7.8	0.018M
	76.0 (71.0 – 82.0)	79.0 (75.0 – 83.0)	
Postoperative neutrophil/lymphocyte ratio (n=321)	8.0 ± 6.1	9.9 ± 5.9	0.001M
	6.6 (4.0 – 8.8)	8.6 (6.0 – 12.5)	
Fasting blood glucose (n=275)	146.7 ± 53.9	121.7 ± 58.0	< 0.001M
	132.0 (103.0 – 197.7)	100.9 (88.1 – 131.8)	
Preoperative critical state			0.010Q
Yes	4 (7.7)	3 (1.0)	
No	48 (92.3)	283 (99.0)	
Postoperative hospital stay (days) (n=322)	9.7 ± 13.2	12.6 ± 9.0	< 0.001M
	4.5 (2.0 – 12.0)	9.0 (7.0 – 14.0)	
Mechanical ventilation time (hours) (n=322)	89.2 ± 154.3	30.4 ± 60.7	< 0.001M
	30.2 (14.5 – 55.7)	12.5 (9.7 – 26.5)	
Reintubation (n=332)			0.001Q
Yes	12 (26.1)	19 (6.6)	
No	34 (73.9)	267 (93.4)	
Mild or transient ARF (n=332)			< 0.001Q
Yes	17 (37.0)	10 (3.5)	
No	29 (63.0)	276 (96.5)	
Surgical infection (any infection within 30 days) (n=332)			< 0.001Q
Yes	17 (37.0)	31 (10.8)	
No	29 (63.0)	255 (89.2)	
CPB time (minutes) (n=318)	99.7 ± 61.2	75.2 ± 29.4	0.007M
	86.0 (69.0 – 110.8)	70.0 (55.0 – 90.0)	
Aortic cross-clamping time (minutes) (n=317)	60.4 ± 38.0	45.8 ± 22.6	0.016M
	56.5 (36.2 – 75.0)	45.0 (27.0 – 60.0)	

^Q^Chi-square test, ^M^Mann-Whitney test

ARF=acute renal failure; CPB=cardiopulmonary bypass; EuroSCORE=European System for Cardiac Operative Risk Evaluation; ICU=intensive care unit

The variables studied were: death; sex; age; mortality risk according to EuroSCORE II (low, medium, high) and in percentage; morbidity (> 7 days of hospitalization or > 4 days in the ICU); weight; extracardiac arteriopathy; previous cardiac surgery; preoperative serum creatinine, serum creatinine at ICU entry, and serum creatinine at ICU discharge; preoperative lymphocytes (total value and %); lymphocytes in the IPO (total value and %); preoperative segmented neutrophils (total value and %); segmented neutrophils in the IPO (total value and %); preoperative band neutrophils (total value and %); band neutrophils in the IPO (total value and %); neutrophil/ lymphocyte ratio in the IPO; fasting glucose; active endocarditis; preoperative critical state; unstable angina; left ventricular function (ejection fraction > 50; 30 to 50; < 30); recent acute myocardial infarct (AMI); previous use of urgency/emergency services; rehospitalization; mechanical ventilation time; reintubation; mild or transient postoperative acute renal failure (ARF); postoperative stroke; surgical infection; cardiopulmonary bypass (CPB) time; aortic cross-clamping time; and return to the ICU.

Patients were analyzed by comparing the variables established with the outcomes death, hospital stay, and ICU stay.

### Ethical Considerations

Data were collected anonymously from the medical records of patients undergoing cardiac surgery with CPB. The research was authorized by the Research Ethics Committee of Faculdade Ciências Médicas de Minas Gerais, under the registration CAAE 54495121.0.0000.5134.

### Sample Calculation

The sample size found for the study was 132 patients. The confidence level adopted was 95%. The margin of error admitted was 5%.

### Statistical Analysis

Categorical variables were presented as absolute and relative frequencies and numerical variables as mean ± standard deviation and median (1st quartile – 3rd quartile). Comparisons of numerical variables between two groups were performed by the Mann-Whitney test and between three groups by the Kruskal-Wallis test with multiple comparisons by the Nemenyi test. The associations between categorical variables were evaluated by the Chi-square test and the correlations between numerical variables by the Spearman’s correlation coefficient. The variables EuroSCORE II, lymphocytes in the postoperative period, and lymphocytes in the postoperative period (%) were evaluated as predictors of mortality through receiver operating characteristic curves and their respective areas under the curve. The analyses were performed using the R software, version 4.0.3, and a significance level of 5% was considered.

## RESULTS

Data from 338 patients over 18 years of age and operated consecutively at the Hospital Universitário Ciências Médicas from January 2015 to December 2017 were evaluated.

### Comparison of Variables with Mortality

The variables sex, age, EuroSCORE II, morbidity (> 7 days of hospitalization or > 4 days in ICU), preoperative creatinine, creatinine at ICU entry, creatinine at ICU discharge, lymphocytes (total and %), segmented neutrophils (%) and neutrophil/lymphocyte ratio in the IPO, fasting glycemia, preoperative critical status, postoperative hospital stay, mechanical ventilation time, reintubation, mild or transient ARF, surgical infection, CPB, and aortic cross-clamping time showed statistically significant results (Table 1).

The value of lymphocytes in the IPO > 2175.0/mm^3^ showed a higher correlation with death than values below this cut, with a *P*-value < 0.001.

The variables weight, presence of extracardiac arteriopathy, previous cardiac surgery, preoperative lymphocytes (total and %), preoperative segmented neutrophils (total and %), postoperative segmented neutrophils (total), preoperative band neutrophils (total and %), postoperative band neutrophils (total and %), presence of active endocarditis, unstable angina, left ventricular function, recent AMI, previous use of urgency/emergency services, re-hospitalization, postoperative stroke, ICU stay in days, and return to the ICU in days did not present statistically significant results, with *P*-value > 0.05.

### Comparison of Variables with Postoperative Hospital Stay in Days

The postoperative hospital stay was significantly correlated with death, EuroSCORE II, EuroSCORE II mortality risk, morbidity, active endocarditis, previous use of urgency/emergency services, mechanical ventilation time, reintubation, mild or transient ARF, postoperative stroke, surgical infection, ICU stay, and return to the ICU, all variables with *P*-value ≤ 0.05.

### Comparison of Variables with Intensive Care Unit Stay in Days

The length of ICU stay was significantly correlated with age, EuroSCORE II, EuroSCORE II mortality risk, morbidity, preoperative creatinine, creatinine at ICU entry, postoperative hospital stay, mechanical ventilation time, reintubation, mild or transient ARF, postoperative stroke, surgical infection, and CPB time, all variables with *P*-value ≤ 0.05.

## DISCUSSION

In this study, the increase in lymphocyte value in the IPO > 2175.0/mm^3^ (± 1481.5) or 14.1% (± 6.7%) (*P*-value < 0.001) proved to be an indicator of postoperative mortality. This data suggests that these patients have an exacerbated inflammatory process, possibly mediated by interleukin-6 (IL-6), altering the hemodynamic behavior of patients.

Leukocytes are the main blood cells involved in the inflammatory response, with neutrophils being the most important in their pathogenesis, followed by lymphocytes^[8]^. These cells, in the acute phase of inflammation, release pro-inflammatory cytokines, such as tumor necrosis factor α (TNF-α) and interleukins (IL-1, IL-6, IL-8), which are endogenous mediators of adhesion molecules, a mechanism that amplifies the sequence of the inflammatory cascade^[8]^. In CPB, this response is initially provoked by the contact of the blood with the synthetic material of its circuit, which is identified as an aggressor by the organism^[8,9,10]^. Added to this, the surgical trauma itself, ischemia, and reperfusion of organs are also triggering factors. Despite the purpose of self-defense, often the response becomes exacerbated, which hemodynamically unbalances the patient. For this reason, the risk of complications and morbidity and mortality increases^[8,9,10]^.

The inflammatory response is triggered by the activation of several humoral and cellular systems, which include coagulation factors, fibrinolysis, the complement system, and cellular components (endothelial cells, lymphocytes, monocytes, neutrophils, and platelets), in addition to endotoxins^[10]^. This reaction also influences the production of oxygen-free radicals and nitric oxide^[9]^. The sequence of these events is responsible for loss of vascular tone, leakage of capillary fluid, and extravasation of leukocytes to the tissues. Clinically, all these summed alterations may present with impaired pulmonary, renal, cerebral, and cardiac functions, in addition to the presence of fever, tachycardia, hypotension, marked edema, coagulopathies, susceptibility to infections, and hemolysis^[11]^.

The release of oxygen-free radicals in the reperfusion process after the use of CPB, at high levels, causes lesions on the deoxyribonucleic acid (DNA) of cells, as well as other structures, resulting in responses such as proliferation, growth arrest, senescence, and even cell death^[9]^. Its effects generate damage to myocardial microcirculation, together with the action of TNF-α, IL-1, and the aggregation of neutrophils to the endothelium^[12]^.

Studies show that serum levels of interleukins, mainly IL-6, are related to morbidity and mortality and can be an accurate marker of inflammatory intensity^[12,13]^. IL-6 is produced by monocytes, endothelial cells, and lymphocytes, being able to represent the degree of active inflammation, and IL-6 level could demonstrate the prognosis of septic shock and the evolution of the patient’s condition^[12]^.

Several therapeutic strategies, both technical and pharmacological, have already been studied to attenuate or even suppress the effects of CPB. However, due to the complexity and synergism of the immense inflammatory cascade that is activated, these mechanisms and their effects still need to be better known^[13]^. The interpretation of these data should take into account the existence of specificities of the inflammatory and immunological response that may vary between each patient^[14]^.

With data from 338 patients, we were able to expand the sample of the previous study, carried out by Ribeiro IVCP et al.^[7]^ (2022), which also demonstrated a correlation between the increased value of lymphocytes in the IPO and mortality, verifying that this marker remained significant in a larger population. There are still few studies in the scientific literature that associate the increased value of lymphocytes in the IPO with a worse prognosis of patients undergoing CVS. Some authors have already demonstrated that the elevation of the neutrophil/lymphocyte ratio, obtained by dividing the absolute neutrophil count by the absolute lymphocyte count, is a finding of significant association with increased short- and long-term mortality, length of hospital and ICU stay, and length of mechanical ventilation^[15,16]^.

No new preoperative markers were identified that could demonstrate a relationship with the prognosis of patients beyond those already cited in the literature. Although the variables weight, extracardiac arteriopathy, previous cardiac surgery, active endocarditis, unstable angina, left ventricular function, and recent AMI were considered risk factors by the EuroSCORE II, these variables did not present statistical relevance in the present study. The other factors with statistical relevance were in agreement with EuroSCORE II^[2]^.

The correlation of preoperative fasting glucose > 146.7 mg/dl (standard deviation ± 53.9) with the occurrence of death was also identified. In the literature, it is recommended that preoperative blood glucose is < 180 mg/dL^[17]^. Authors report that worse glycemic control is associated with adverse outcomes and that elevated intra and postoperative values increase the risk of morbidity and mortality^[18,19,20]^.

### Limitations

Among the limitations of this study, it is necessary to mention that it was carried out with a single-center sample from a hospital that usually attends patients in serious conditions. In addition, all patients were operated by the same team of surgeons. Furthermore, although the finding of postoperative lymphocytes is statistically significant, it has a very wide standard deviation (2175.0 ± 1481.5), which overlaps with the data from the group of postoperative lymphocytes with no death (1447.1 ± 804.2).

Because of these limitations, in order to reduce possible biases, to find more accurate results, and to achieve more important validation, it is necessary to extend the research to a multicenter study, so that a larger sample size with diverse characteristics is included. The perspective is that, with the knowledge that this variable can predict the risk of patients in the IPO, it is possible to institute treatments that act earlier, minimizing complications and mortality. The study of inflammatory mechanisms in CVS with CPB opens possibilities to establish new approaches in the postoperative period.

## CONCLUSION

The value of lymphocytes in the postoperative period > 2175.0/ mm3 is an indicator of poor prognosis in the sample studied.

In addition, the variables female sex, age, high level of EuroSCORE II, increased stays in the ICU and in the ward, elevation of creatinine in the preoperative period and at discharge from the ICU, elevation of the percentage of IPO segmented neutrophils, high IPO neutrophil/ lymphocyte ratio, fasting hyperglycemia, preoperative critical condition, reintubation, mild or transient ARF, surgical infection, CPB, aortic cross-clamping, and mechanical ventilation durations also had an impact on the mortality outcome.

## Abbreviations, Acronyms & Symbols

ARF: Acute renal failure
AMI: Acute myocardial infarct
CPB: Cardiopulmonary bypass
CVS: Cardiovascular surgeries
EuroSCORE: European System for Cardiac Operative Risk Evaluation
ICU: Intensive care unit
IL-6: Interleukin 6
IPO: Immediate postoperative period
TNF-α: Tumor necrosis factor α
